# HS-543 induces apoptosis of Imatinib-resistant chronic myelogenous leukemia with T315I mutation

**DOI:** 10.18632/oncotarget.2837

**Published:** 2014-12-02

**Authors:** Soo Jung Kim, Kyung Hee Jung, Hong Hua Yan, Mi Kwon Son, Zhenghuan Fang, Ye-Lim Ryu, Hyunseung Lee, Joo Han Lim, Jun-Kyu Suh, JinHee Kim, Soyoung Lee, Sungwoo Hong, Soon-Sun Hong

**Affiliations:** ^1^ College of Medicine, Inha University, Sinheung-dong, Jung-gu, Incheon, Republic of Korea; ^2^ National Research Center for Sexual Medicine, College of Medicine, Inha University, Sinheung-dong, Jung-gu, Incheon, Republic of Korea; ^3^ Center for Catalytic Hydrocarbon Functionalizations, Institute for Basic Science (IBS), Republic of Korea; ^4^ Department of Chemistry, Korea Advanced Institute of Science and Technology (KAIST), Daejeon, Republic of Korea

**Keywords:** HS-543, Bcr-Abl, T315I, Chronic Myeloid Leukemia

## Abstract

Chronic myeloid leukemia (CML) is characterized by a constitutive activation of Bcr-Abl tyrosine kinase. Bcr-Abl/T315I is the predominant mutation that causes resistance to Imatinib. In the present study, we synthesized a novel Bcr-Abl inhibitor, HS-543, and investigated its effect on cell survival or apoptosis in CML cells bearing Bcr-Abl/T315I (BaF3/T315I) or wild-type Bcr-Abl (BaF3/WT). HS-543 showed anti-proliferative effects in the BaF3/WT cells as well as the BaF3/T315I cells with resistance to Imatinib and strongly inhibited the Bcr-Abl signaling pathway in a dose-dependent manner. Furthermore, it significantly increased the sub G_1_ phase associated with early apoptosis, with increased levels of cleaved PARP and cleaved caspase-3, as well as the TUNEL-positive apoptotic cells. In addition, we found that HS-543 induced apoptosis with the loss of mitochondrial membrane potential by decreasing the expression of Mcl-1 and survivin, together with increasing that of Bax. In BaF3/T315I xenograft models, HS-543 significantly delayed tumor growth, unlike Imatinib. Our results demonstrate that HS-543 exhibits the induction of apoptosis and anti-proliferative effect by blocking the Bcr-Abl signaling pathway in the T315I-mutated Bcr-Abl cells with resistance to Imatinib. We suggest that HS-543 may be a novel promising agent to target Bcr-Abl and overcome Imatinib resistance in CML patients.

## INTRODUCTION

Chronic myeloid leukemia (CML) is a clonal myeloproliferative disorder that is characterized by high levels of immature white blood cells, which is one of the most understood neoplasms. Annually, newly diagnosed cases of CML in the United States is estimated to be about 4,800 to 5,200. The estimated prevalence of CML cases in the United States was approximately 25,000~30,000. CML is defined by the formation and presence of the Philadelphia (Ph) chromosome, which results from the reciprocal chromosomal translocation t(9;22) and is represented as Bcr–Abl fusion gene. This Bcr–Abl fusion protein is found in ~95% of patients with CML and 20~30% of adult patients with acute lymphoblastic leukemia (ALL) [1], a lesser extent Bcr-Abl+ ALL [2]. The Bcr–Abl aberrant tyrosine kinase is mainly responsible for malignant transformation by activating multiple signal transduction pathways including Stats, MAPK/Erk, and PI3K/Akt, which ultimately lead to increased survival, proliferation, and escape from apoptosis [3, 4]. For this reason, Bcr–Abl tyrosine kinase has been considered as an important target for CML therapeutics. Meanwhile, Imatinib is considered as the first selective Bcr–Abl tyrosine kinase inhibitor (TKI) for cancer therapy, which interrupts Bcr–Abl oncogenic signaling. Therefore, Imatinib has become the new “gold standard” for the treatment of patients with CML. Especially, 80% of newly diagnosed patients with chronic-phase CML has shown a complete cytogenetic response to treatment with Imatinib over a median follow-up of 54 months. Although the initial response rates are high, Imatinib fails in up to 40% of patients because of disease resistance or Bcr-Abl kinase domain mutations, and unacceptable side effects. To circumvent the resistance, more potent TKIs such as Nilotinib and Dasatinib as the second generation TKIs, have been approved. However, these compounds do not show therapeutic activities against all Imatinib-resistant mutants of Bcr-Abl, and finally a long-term tolerablility problem has emerged.

Drug resistance during the Imatinib treatment is mostly related to point mutations occurring within the kinase domain of Bcr-Abl. To date, over 90 different point mutations in the Bcr-Abl kinase domain have been isolated from relapsed CML patients, who are resistant to Imatinib. Among those, the T315I is the most stubborn point mutation having an impact on the binding of Imatinib with Bcr-Abl kinase domain. Also, the T315I is responsible for approximately 15% of the cases of relapse in CML and Bcr-Abl+ ALL patients on Imatinib therapy [5], and there is high occurrence of E255K and M351T mutations [6, 7]. Most mutations, except T315I, may be eradicated with the appropriate choice and combinations of second generation tyrosine kinase inhibitors. However, there is still no effective TKI available for CML with the T315I mutation. Considering these facts, the T315I mutation remains a crucial clinical challenge, and it is imperative to develop novel strategies to overcome this resistance.

To resolve these problems, we synthesized and screened a chemical library of novel series of benzothiazole-based inhibitors that are effective against wild-type and T315I mutant Bcr-Abl kinases [8]. Of the benzothiazole-based inhibitors, 1-(6-(2-methoxyphenyl)benzo[d]thiazol-2-yl)-3-(2-(4-methylpiperazin-1-yl)ethyl), HS-543 was identified as the potent Bcr-Abl kinases inhibitor. Here, we investigated whether or not HS-543 possesses anti-cancer activity in BaF3/T315I or BaF3/WT and the molecular mechanisms involved in this process. In this study, our results showed that HS-543 suppressed the Bcr-Abl pathway and induced apoptosis in not only BaF3/WT cells but also T315I-mutated Bcr-Abl cells with resistance to Imatinib.

## RESULTS

### Synthesis of HS-543 and its binding mode to Bcr-Abl

**Figure 1:**
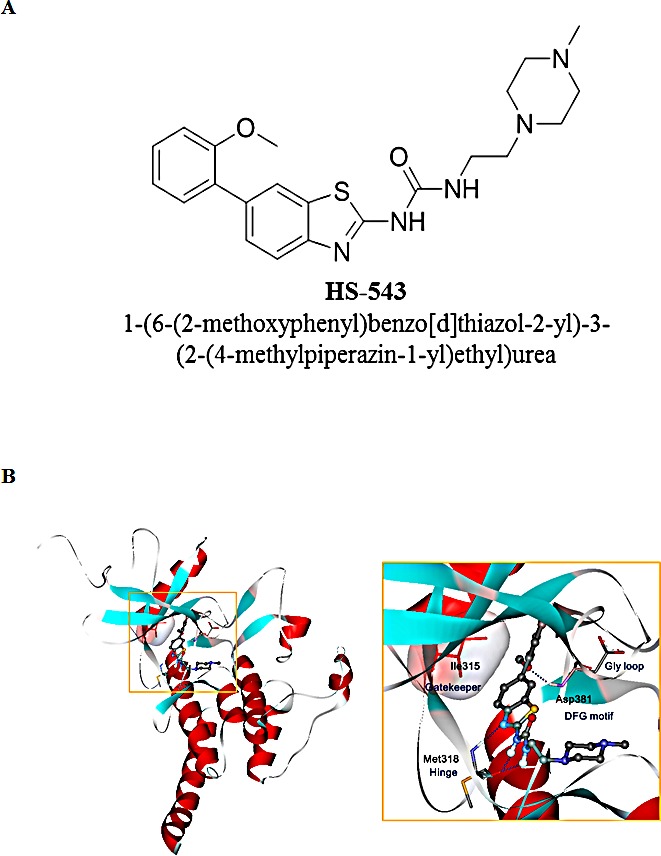
Structure and predicted binding mode of HS-543 with Abl/T315I (A) Structure of HS-543, 1-(6-(2-methoxyphenyl)benzo[d]thiazol-2-yl)-3-(2-(4-methyl piperazin-1-yl)ethyl)urea. (B) The putative binding mode of HS-543 in the Bcr-Abl model.

We identified a novel Bcr-Abl inhibitor, 1-(6-(2-methoxyphenyl)benzo[d]thiazol-2-yl)-3-(2-(4-methylpiperazin-1-yl)ethyl) urea or HS-543. As shown in Table 1, HS-543 was further subjected to kinase selectivity profiling against a panel of cancer-related kinases at 1 μM concentrations in a high-throughput binding assay (KINOMEscan^®^, Ambit Biosciences, San Diego, CA). Percent of control (POC) determinations were also performed. HS-543 displayed only a high degree of selectivity to Abl but not to other kinases such as Aurora and c-Kit, which are known to have high structural similarities to Abl. The only kinases against which HS-543 demonstrated target activity were Abl (POC 1.6) and Abl/T315I (POC 3.1). We prepared the receptor model in DFG-in conformation from the X-ray crystal structure of T315I mutant in a complex with PPY-A (PDB code: 2Z60) [9]. Fig. 1 shows the possible binding mode of HS-543 in the ATP binding site of T315I mutant Abl, which was obtained through a docking simulation using the Autodock program [10]. The docking simulation indicated that C6 methoxyphenyl moiety was surrounded by a hydrophobic pocket, and methyl piperazine moiety in C2 urea was expected to the solvent front. In the hinge region, the nitrogen of benzothiazole scaffold and the C2 urea group appeared to form a tight hydrogen bond with the backbone residue of Met318 in the hinge region. Especially, both of the two NHs in the distorted C2 urea group appeared to act as hydrogen bond donors and form hydrogen bonds with aminocarbonyl oxygen of the Met318 backbone. C6 aryl moiety was directed toward the region nearby the gatekeeper and catalytic lysine. The C6 phenyl ring seemed to form favorable van der Waals interactions with the larger mutated gatekeeper, Ile318, without causing steric hindrance. In addition, the oxygen of the 2′-methoxy group in the C6 phenyl ring could contribute to an additional hydrogen bond with the Asp381 backbone.

**Table 1:** High-throughput binding assay against a panel of kinases A panel of 96 kinases was tested at 1 μM concentrations in a high-throughput binding assay. Only representative kinases are shown here. Lower percent of control (POC) values represent stronger hits. Values shown are the mean of duplicate measurements

KINOME scan Gene Symbol	Entrez Gene Symbol	POC	Compound Concentration (nM)
**ABL1(T315I)-phosphorylated**	**ABL1**	**3.1**	**1000**
**ABL1-phosphorylated**	**ABL1**	**1.6**	**1000**
AKT1	AKT1	94	1000
AURKA	AURKA	100	1000
BRAF	BRAF	92	1000
CDK11	CDK19	91	1000
EGFR	EGFR	87	1000
ERK1	MAPK3	100	1000
FAK	PTK2	100	1000
FGFR1	FGFR1	80	1000
GSK3B	GSK3B	100	1000
IKK-beta	IKBKB	100	1000
JAK1(JH1domain-catalytic)	JAK1	100	1000
KIT	KIT	58	1000
KIT(D816V)	KIT	86	1000
MEK1	MAP2K1	100	1000
MET	MET	74	1000
P38-alpha	MAPK14	100	1000
PAK1	PAK1	88	1000
PIK3CA	PIK3CA	100	1000
PIM1	PIM1	98	1000
PLK1	PLK1	88	1000
ROCK1	ROCK1	100	1000
TRKA	NTRK1	95	1000

### HS-543 inhibits the proliferation of leukemia cells

**Figure 2:**
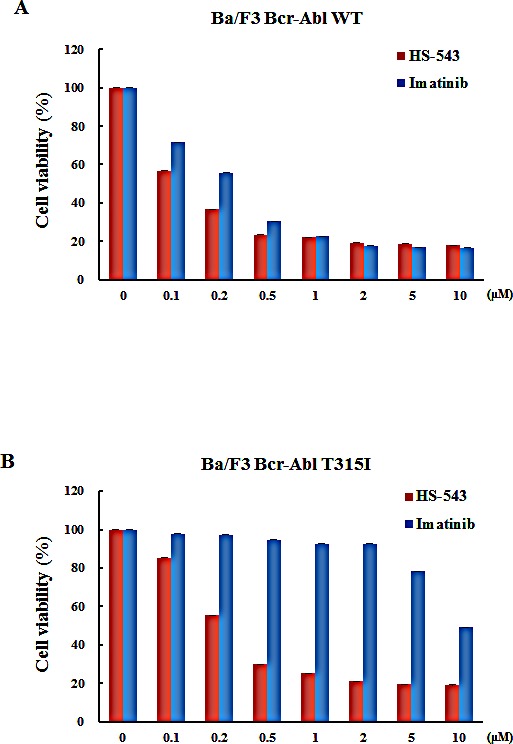
Cytotoxic effect of HS-543 in leukemia cells Cytotoxic effects of leukemia cells treated with HS-543 and Imatinib were measured using the MTS assay. Cells were seeded in 96-well culture plates, and were treated with various concentrations of HS-543 and Imatinib. Cells were subjected to an MTS assay following incubation for 48 h. Data are represented as mean ± S.D. from triplicate wells.

MTS assays were performed to evaluate the effect of HS-543 on the growth of BaF3/WT and BaF3/T315I cells. The cells were treated with various concentrations of HS-543 and Imatinib for 48 h. As shown in Fig. 2A, both HS-543 and Imatinib treatments reduced the cell viability of the BaF3/WT cells; IC_50_ values were 0.33 μM for Imatinib and 0.116 μM for HS-543. As shown in Fig. 2B, HS-543 significantly reduced the cell viability of BaF3/T315I cells (IC_50_ = 0.33 μM), while Imatinib showed little effect (IC_50_ = 10 μM). These data suggest that HS-543 exhibits the potent inhibitory activity in T315I mutated Bcr-Abl cells with the resistance to Imatinib, as well as Bcr-Abl expressing leukemic cells.

### HS-543 inhibits Bcr-Abl signaling pathway in BaF3/T315I cells

**Figure 3:**
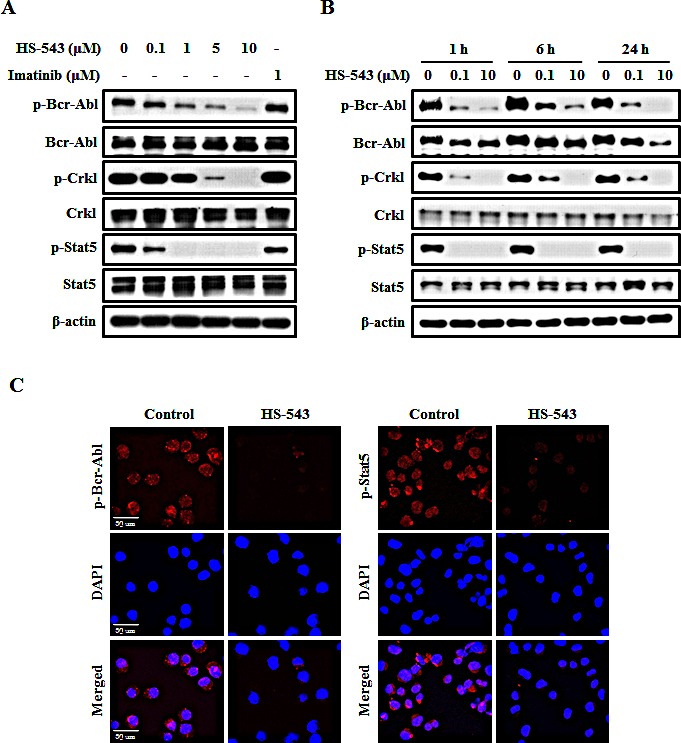
Effect of HS-543 on the Bcr-Abl signaling pathway in BaF3/T315I cells (A) Cell were treated with HS-543 (0.01–10 μM) or Imatinib (1 μM) for 24 h. Western blot experiments for Bcr-Abl, Crkl, and Stat5, and the respective phosphorylated proteins were performed with cell lysates. (B) Cells were treated with HS-543 for time-dependent manner. (C) After BaF3/T315I cells were treated with 10 μM HS-543 for 3 h, p-Bcr-Abl and p-Stat5 levels were detected by confocal fluorescent microscopy (200× magnification).

To test if the antiproliferative effects of HS-543 were dependent on the inhibition of Bcr-Abl activity, phosphorylation of Bcr-Abl and its respective downstream signals, Crkl and Stat5, were measured by western blotting. As shown in Fig. 3A and 3B, HS-543 strongly inhibited the phosphorylation of Bcr-Abl (Tyr^177^) in BaF3/T315I cells. Likewise, the phosphorylation levels of Crkl (Tyr^207^) and Stat5 (Tyr^694^) were effectively suppressed in a dose- and time-dependent manner. In contrast, Imatinib did not alter the phosphorylation levels of Bcr-Abl, Crkl and Stat5 in BaF3/T315I cells. These results were confirmed by a confocal fluorescent microscopy (Fig. 3C).

### HS-543 induces apoptotic cell death in BaF3/T315I cells

**Figure 4:**
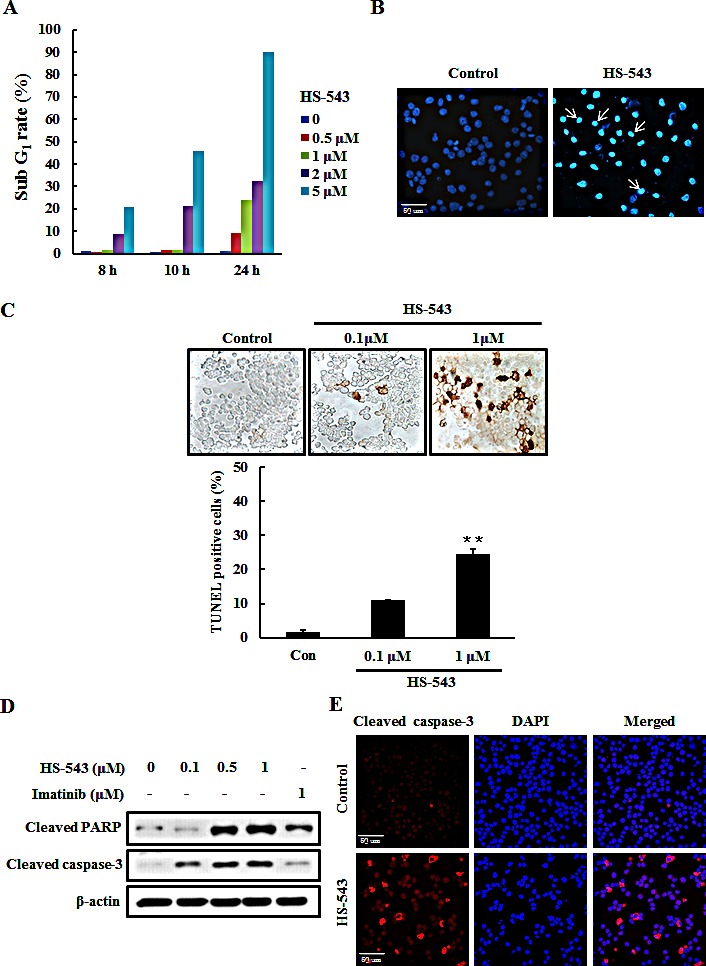
Effect of HS-543 on apoptosis of BaF3/T315I cells (A) Cells were incubated with HS-543 for various dose and times. Control and treated cells were collected, stained with PI, and analyzed by flow cytometry. (B) Cell were treated with HS-543 (1 μM) for 24 h. Then, cells were fixed in ice-cold 4% PFA, washed with PBS, and then stained with 1 μg/ml Hoechst 33342 for 20 min at room temperature. The stained cells were examined under immunofluorescence for evidence of nuclear fragmentation (400× magnification). (C) Cells were treated with HS-543 for 24 h and were performed with TUNEL assay. Data are represented as mean ± S.D. from triplicate wells. **p < 0.001 as compared to control. (D) For detection of expression of cleaved PARP and cleaved caspase-3, the cells were treated with HS-543 and Imatinib for 36 h. (E) (E) Immunofluorescence of cleaved caspase-3 after treatment of HS-543 (1 μM) in BaF3/T315I cells for 24 h (400× magnification).

In order to determine whether the anti-cancer effect of HS-543 in BaF3/T315I cells was associated with the induction of apoptosis, we performed several cell-based apoptosis assays. We first assessed the cell cycle distribution by a flow cytometric analysis. The cells were collected, stained with PI, and analyzed by flow cytometry after they were incubated with HS-543 for various doses and times. As shown in the Fig. 4A, HS-543 increased the number of cells in the sub G_1_ phase, associated with early apoptosis without changes of the cell cycle arrest. To characterize nuclear morphology, the nuclei were stained with Hoechst, and a TUNEL assay was performed. As a result, the cells treated with HS-543 were presented with more prominent morphological features of apoptotic cells, such as bright nuclear condensation by Hoechst staining (Fig. 4B). HS-543-induced apoptosis was confirmed by detecting DNA fragmentation using TUNEL staining. Cells were treated with HS-543 for 24 h (Fig. 4C, **p <0.001). Furthermore, HS-543 significantly increased the expression of cleaved PARP and cleaved caspase-3, apoptosis-related molecules in BaF3/T315I cells (Fig. 4D), compared with Imatinib. An increase of cleaved caspase-3 was also confirmed by immunofluorescence after treating with 1 μM HS-543 in BaF3/T315I cells for 24 h (Fig. 4E).

### HS-543 induces mitochondria-dependent apoptosis in BaF3/T315I cells

**Figure 5:**
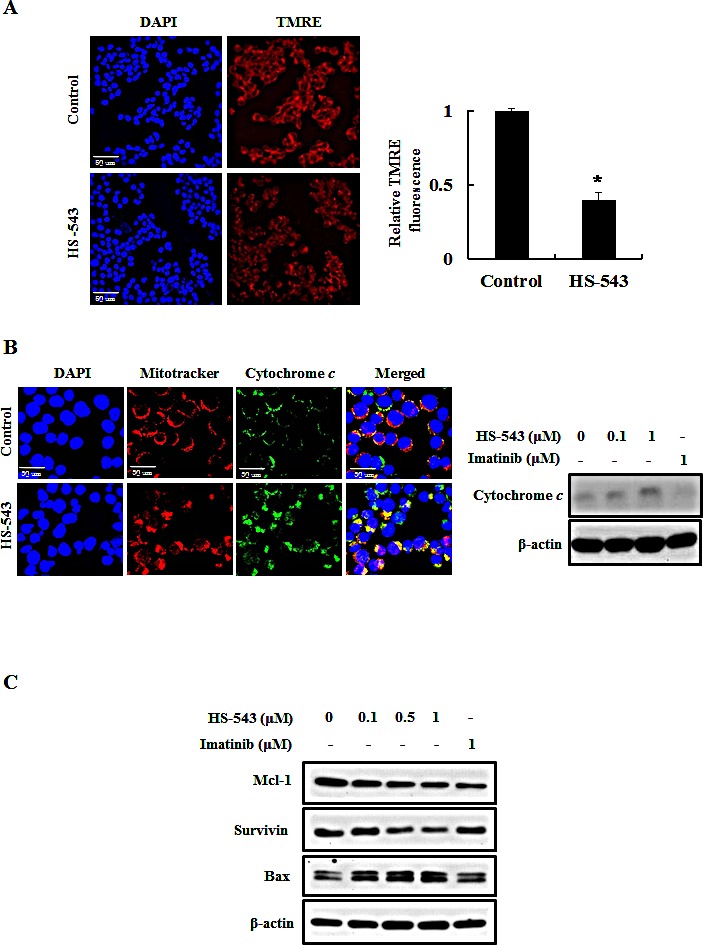
Effect of HS-543 on mitochondria-related apoptosis of BaF3/T315I cells (A) BaF3/T315I cells were treated with HS-543 at 1 μM for 12 h and were stained with TMRE for 30 min at 37°C and analyzed with a confocal fluorescent microscopy (400× magnification). *p < 0.01 as compared to control. (B) BaF3/T315I cells were treated with HS-543 at 1 μM for 10 h and were stained with mitotracker and cytochrome *c*. Localization of cytochrome *c* in the cytosol by HS-543 was analyzed with a confocal fluorescent microscopy (1200× magnification). For western blotting, BaF3/T315I cells were treated with 0.1 and 1 μM HS-543 together with 1 μM Imatinib for 12 h. (C) The expression of Mcl-1, survivin and Bax were assayed by western blot in BaF3/T315I cells. Cells were treated with HS-543 and Imatinib for 12 h.

Loss of mitochondrial membrane potential (MMP) induces mitochondrial permeability transition and cytosolic translocation of apoptotic proteins [11]. Thus, we measured MMP and apoptosis in HS-543-treated BaF3/T315I cells using TMRE. As shown in Fig. 5A, HS-543 significantly reduced the fluorescence intensity reflecting MMP (*p < 0.01). Since MMP can trigger the release of mitochondrial cytochrome *c* into the cytosol and induces mitochondria-mediated protein families such as Mcl-1, survivin and Bax, we investigated their expression by HS-543 in BaF3/T315I cells [9]. As shown in Fig. 5B, we observed that the treatment of HS-543 increased cytochrome *c* release by immunostaining and western blotting. In addition, HS-543 increased the expression of Bax and decreased the expression of the anti-apoptotic proteins survivin and Mcl-1 (Fig. 5C). These results showed that HS-543 induced apoptosis through change of mitochondria-related proteins in BaF3/T315I cells.

### HS-543 inhibits tumor growth in mouse xenograft models

**Figure 6:**
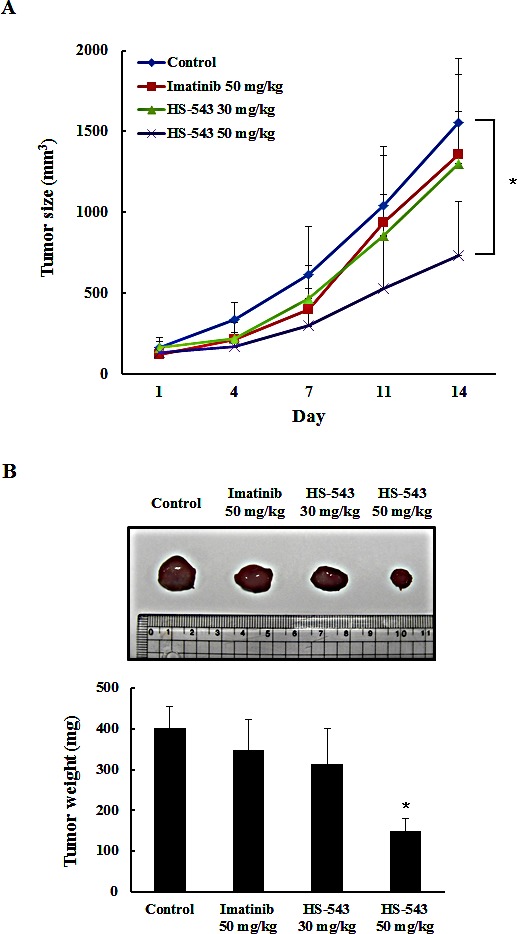
*In vivo* anticancer effect of HS-543 in mouse xenograft model (A) Tumor growth of BaF3/T315I xenograft in BALB/c nude mice. All mice were subjected to implantation in the flank by a subcutaneous injection of BaF3/T315I (1×10^5^ cells/200 μL PBS). HS-543 (30 and 50 mg/kg) and Imatinib 50 mg/kg was intraperitoneally injected 5 times a week for 14 days. Tumor size was measured every 2 days. Data were represented as mean ± S. D. (n = 5). (B) After mouse sacrifice, representative photographs and mean weights of the isolated tumors. *p < 0.01 as compared to control.

**Figure 7:**
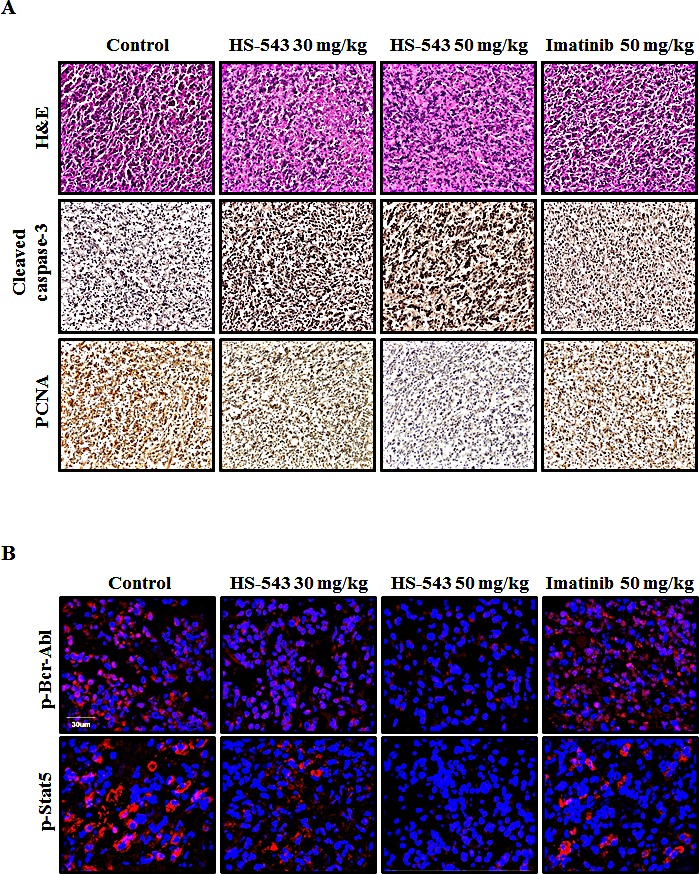
Effect of HS-543 on proliferation and apoptosis in isolated tumor from mouse xenograft model (A) Tumors were excised and processed for immnunohistochemistry to detect cleaved caspase-3 and PCNA including H&E staining. (B) Tumors were stained with p-Bcr-Abl and p-Stat5 and were detected by confocal fluorescent microscopy (400× magnification).

We extended our study to an *in vivo* mouse xenograft model. After inoculation with BaF3/T315I cells, mice were intraperitoneally injected with HS-543 at doses of 30 and 50 mg/kg, and Imatinib at a dose of 50 mg/kg 5 times a week for 14 days. While Imatinib treatment didn't show significant anticancer effect in this BaF3/T315I cell xenograft model, HS-543 potently inhibited the progression of tumor growth and more visible and significant on day 14 as compared with the control group (*p < 0.01, Fig. 6A). Isolated tumor weight was also remarkably lower in the HS-543 treated group than in the control group (Fig. 6B, *p < 0.01). No significant changes in body weight or adverse effect were observed in all groups. To further confirm whether HS-543 inhibits tumor growth through the induction of apoptosis and inhibition of proliferation, we identified the expression of cleaved caspase-3 and PCNA in the isolated tumor tissues. As expected, the treatment with HS-543 increased the expression of cleaved caspase-3 and decreased for PCNA in the HS-543 treated group as compared to the control and Imatinib groups (Fig. 7A). Furthermore, the treatment with HS-543 decreased the phosphorylation of p-Bcr-Abl and p-Stat5; thus, regulating many different events involved in cell survival and proliferation (Fig. 7B). Taken together, these results demonstrate that HS-543 has a potent antitumor efficacy in mouse xenograft model bearing BaF3/T315I cells.

## DISCUSSION

In 95% of CML cases, the product of a reciprocal translocation between chromosomes 9 and 22, the Philadelphia chromosome, is detected, which is characterized by the presence of Bcr–Abl fusion gene, representing a subtype of leukemia with poor prognosis, rapidly acquiring resistance to the Imatinib treatment during therapy [12, 13]. Imatinib is the molecular targeted agent that selectively inhibits the Bcr–Abl tyrosine kinase activity. To date, Imatinib has been recognized as a revolutionary treatment of CML, due particularly to the durable remission most chronic-phase patients experience. Also, Imatinib inhibits Bcr–Abl kinase with 50% inhibitory concentration values of 0.1–0.5 μM in cell-based assays [14, 15]. Targeted therapy to inhibit the oncogene Bcr-Abl seems to be somewhat successful. However, Imatinib resistance often occurs in patients especially in CML accelerated phase and blast crisis, and almost unrelievedly occurs in patients with the Bcr-Abl expression, showing shortcomings of the therapy. According to Imatinib resistance, amplification and mutation of Bcr-Abl is believed to be the predominant. To overcome this acquired resistance to Imatinib, a lot of researchers have developed a new, second-generation ATP-competitive Abl kinase inhibitors, such as AMN107, Dasatinib, INNO-406 and PD166326, which have stronger affinities for the ATP-binding site of Bcr-Abl kinase than Imatinib, and has shown partial effect for Imatinib-resistant patients [16-20]. Although these novel inhibitors can effectively inhibit the phosphorylation of the mutated Bcr-Abl (E255K and M351T), they showed little effect on Bcr-Abl/T315I [21]. Therefore, innovative treatment is necessary to over-ride TKI-resistant mutations, or to promote elimination of Bcr-Abl harboring cells in patients. The principal objective of this study is to identify the effective targeted chemotherapy against CML cells carrying T315I-mutant Bcr-Abl that confers resistance to Imatinib. In this study, we demonstrated that HS-543, a novel Bcr-Abl TKI, had high affinity for the ATP-binding site, resulting in high specificity to BaF3/T315I (Table 1). In light of these results, we investigated whether HS-543 had potent activity on Bcr-Abl target inhibition in BaF3/WT and BaF3/T315I CML cell lines. HS-543 potently inhibited the proliferation in both BaF3/WT (IC_50_ = 0.116 μM) and BaF3/T315I (IC_50_ = 0.33 μM) cells. However, Imatinib did not inhibit the proliferation in BaF3/T315I cells, since IC_50_ values were more than 10 μM.

Bcr-Abl activates multiple downstream signaling pathways, including Stat5 pathway, which contribute to leukemic cell proliferation and survival [22, 23]. Also, the Stat5 activation is mediated by the adaptor protein Crkl [24, 25]. In addition, Stat5 activity appears to play a major role in the antiapoptotic and proliferative abilities of Bcr-Abl transformed cells [22, 23]. Previous studies have established phosphorylation of Stat5 as an indicator of Bcr-Abl activity [26]. Therefore, we identified whether HS-543 could inhibit Bcr-Abl signaling pathway. As expected, HS-543 distinctly inhibited the phosphorylation of Bcr-Abl, which indicates decreased Abl kinase activity, as well as a pronounced inhibition of Bcr-Abl downstream target Stat5 phosphorylation in BaF3/T315I cells. Also, phosphorylation of another well-known Bcr-Abl downstream target Crkl was obviously reduced after the treatment with HS-543. Interestingly, Imatinib inhibited the phosphorylation of Bcr-Abl pathways, such as Bcr-Abl, Stat5, and Crkl in BaF3/WT cells, but not in BaF3/T315I cells. These results revealed that the decrease of phosphorylation of Crkl and Stat5 by HS-543 in the cells with the highly resistant mutation T315I was significant, which indicate an effective inhibition of Bcr-Abl carrying this highly resistant mutation.

Also, HS-543 induces mitochondrial apoptosis in BaF3/T3151 cells. In fact, apoptosis has been noticed to be associated with a loss of mitochondrial membrane potential, which may correspond to the opening of an outer membrane pore, leading to the release of cytochrome *c* into the cytoplasm [27]. Also, the release of cytochrome *c* induces the expression of various pro-apoptotic proteins in cytoplasm [28]. Especially, Bax initiates a mitochondrial permeability shift and induces apoptosis by moving from the cytosol to the mitochondrial membrane [29]. And, Mcl-1 and survivin as anti-apoptotic proteins have been reported to bind to caspases to inhibit apoptosis signaling [30]. More importantly, Bcr-Abl inhibits apoptosis through regulating Bcl-2 family members [31], increasing the expression of antiapoptotic Bcl-2 family members, such as Bax and Bcl-2 through activation of the transcription factor Stat5 [32-34]. Additionally, Bcr-Abl has been shown to prevent mitochondrial cytochrome *c* release through a posttranslational mechanism [35]. Accordingly, we assumed that HS-543 would affect the mitochodrial membrane potential and induce apoptosis since HS-543 showed a high specific inhibition against Bcr-Abl. Indeed, we observed that HS-543 increased cytochrome *c* release and decreased the expression of Mcl-1 and survivin, whereas increased the expression of Bax. These changes led to an increase the expression of cleaved caspase-3 and cleavage of PARP. These events were supported by *in vivo* results, showing that HS-543 inhibited the tumor growth and induced apoptosis by increasing the expression of cleaved caspase-3 in tumor tissues of BaF3/T315I cells xenograft mice. However, Imatinib did not change the expression of apoptosis-related molecules *in vitro and in vivo*. Overall, HS-543 showed to induce apoptosis via an intrinsic mitochondria-dependent pathway in BaF3/T315I cells, suggesting that the apoptosis effect by HS-543 may be achieved by inhibition of Bcr-Abl signaling pathways.

In conclusion, our results showed that the novel selective Bcr-Abl TKI, HS-543, had potent anticancer activity against the cells bearing wild type and T315I mutant type Bcr-Abl. Given the mechanism of action and the promising activity of HS-543 against Imatinib-resistant CML cells, our results provide an important vision to be considered for future clinical investigations in CML patients with Imatinib resistance. Here, we suggest that HS-543 may have great potential in overcoming T315I mutation–induced Imatinib resistance.

## METERIALS AND METHODS

### Cells and materials

The BaF3/WT and BaF3/T315I cells were kindly provided by Dr. Deininger (Huntsman Cancer Institute, Salt Lake City, UT), which inducibly expressed wild-type Bcr-Abl and Bcr-Abl with the T315I mutation, respectively. BaF3/WT and BaF3/T315I cells were grown in Roswell Park Memorial Institute Media 1640 (RPMI 1640), containing 10% fetal bovine serum (FBS) and 1% penicillin/streptomycin. RPMI 1640; FBS and penicillin/streptomycin were purchased from GIBCO (Grand Island, NY). Imatinib was purchased from LC laboratories (Woburn, MA), and 3-(4,5-dimethylthiazol-2-yl)-5-(3-carboxymethoxyphenyl)-2-(4-sulfophenyl)-2H-tetrazolium, inner salt (MTS) were purchased from Promega (Madison, WI).

### Enzyme binding assay

The enzyme binding assay of HS-543 was performed against a panel of 96 kinases using KINOMEscan assays at the Ambit Biosciences Corp (San Diego, CA). In brief, HS-543 was screened at 1 μM and results for primary screen binding interactions were reported as ‘% Ctrl’. % Ctrl Calculation = (HS-543 signal − positive control signal / negative control signal − positive control signal) x100, where negative control is DMSO (100% Ctrl), and positive control is control compound (0% Ctrl). Accordingly, lower percent of control (POC) values represent stronger hits [8].

### Cell viability assay

Cell viability of corresponding compounds was determined by MTS assay. The cells were seeded and treated onto 96-well plates at a density of 1 × 10^3^ cells per well and incubated at 37 °C for 48 h. The cells were treated with either HS-543 or Imatinib at the indicated concentrations (0.001–10 μM). Then, 20 μL of MTS labeling mixture (1 mL of MTS/50 μL of phenazine methosulfate [PMS]) was added to each well. After incubation for 4 h, optical density (OD) was determined using a microplate reader by measuring the absorbance at wavelengths of 540 nm.

### Western blotting

After the cells were treated with various concentrations of either HS-543 or Imatinib and incubated at 37°C for various times, they were collected and washed with cold phosphate-buffered saline (PBS). Then, the cells were lysed with a RIPA buffer (BIOSESANG, Korea) containing protease and phosphatase inhibitor cocktails (GenDEPOT, Barker, TX). The proteins were resolved by sodium dodecyl sulfate–polyacrylamide gel electrophoresis (SDS–PAGE), and transferred onto the nitrocellulose membranes. The blots were immunostained with the appropriate primary antibodies followed by secondary antibodies conjugated to horseradish peroxidase. Antibody binding was detected with an enhanced chemiluminescence reagent (Bio-Rad. Hercules, CA). Primary monoclonal antibodies against the following factors were used; p-Bcr-Abl (Tyr^177^), p-Crkl (Tyr^207^), p-Stat5 (Tyr^694^), Bcr-Abl, Crkl, Stat5, cleaved PARP, cleaved caspase-3, cytochrome *c*, survivin, Mcl-1, Bax and β-actin (Cell Signaling Technology, Danvers, MA). Secondary antibodies were purchased from Santa Cruz Biotechnology (Dallas, TX).

### Immunofluorescence

BaF3/T315I cells were plated in 48-well plates with RPMI medium and treated with 1 μM HS-543 for various times. The cells were then suspended on poly-l-lysine–coated slides, followed by Shandon Cytospin 3 (Akribis Scientific, Cheshire, WA) for 3 min at 1000 rpm. Thereafter, the cells were fixed in 4% paraformaldehyde (PFA) for 15 min at room temperature and washed twice with PBS. The cells were blocked in 5% horse and goat serum in PBS for 1 h at room temperature, and then incubated in a humidified chamber at 4°C overnight with primary antibodies including rabbit anti-p-Bcr-Abl (Tyr^177^) and p-Stat5 (Tyr^694^) (Cell Signaling Technology). After washing twice with PBS, the cells were incubated with rabbit tetramethyl rhodamine isothiocyanate (TRITC) secondary antibody (Dianova, Germany) for 1 h at room temperature. They were also stained with 4,6-diamidino-2-phenylindole (DAPI) to visualize the nuclei. The slides were then washed twice with PBS and covered with DAKO (Carpinteria, CA) before viewing with a confocal laser scanning microscope (Olympus, Japan).

Also, after deparaffinization, immunostaining was performed, using 8-μm-thick sections of the tumor samples. The tissue sections were blocked with a normal goat or horse serum (Vector Laboratories. Burlingame, CA) for 1 h, and incubated at 4°C overnight in 1:50 dilutions of p-Bcr-Abl (Tyr^177^) and p-Stat5 (Tyr^694^) (Cell Signaling Technology). After washing twice with PBS, the cells were incubated with rabbit TRITC secondary antibody for 1 h at room temperature. They were also stained with DAPI to visualize the nuclei. The slides were then washed twice with PBS and covered with DAKO before viewing with a confocal laser scanning microscope.

### Cell cycle arrest

BaF3/T315I cells were plated in 10 cm dishes with RPMI medium and treated with HS-543 at the indicated concentrations (0.5–5 μM). The cells were collected and fixed in cold 70% ethanol at −25°C overnight. After washing with PBS, the cells were subsequently stained with 50 μg/mL propidium iodide (PI) and 100 μg/mL RNase A for 30 min at room temperature in the dark; then a flow cytometric analysis was performed to determine the percentage of cells in specific phases of sub G_1_ using a FACS Calibur flow cytometer (BD Biosciences. San Jose, CA). Flow cytometric data was analyzed using FlowJo software (Tree Star. Ashland, OR).

### Hoechst staining and TUNEL staining

BaF3/T315I cells were plated in 48-well plates with RPMI medium and treated with HS-543 (1 μM). The cells were then suspended on poly-l-lysine-coated slides, followed by Shandon Cytospin 3 for 5 min at 1000 rpm. They were then fixed in 4% PFA for 15 min at room temperature, washed with PBS, and stained with 1 μg/mL Hoechst 33342 (Cell Signaling Technology) for 20 min at room temperature. The stained cells were examined under a fluorescence microscope for evidence of nuclear fragmentation. Terminal deoxynucleotidyl transferase-mediated nick end labeling (TUNEL) was subsequently performed using a TUNEL kit (Merck Millipore, Temecula, CA) in accordance to the manufacturer's instructions.

### Measurement of mitochondrial membrane potential

Mitochondrial membrane potential (MMP, Δ*ψm*) was assessed using Multi-Parameter Apoptosis Assay Kit (Cayman, Ann Arbor, MI). BaF3/T315I cells were treated with 1 μM HS-543 for 12 h. The cells were incubated with tetramethylrhodamine ethyl ester (TMRE), and DAPI staining at dark room condition. The cells were then suspended on poly-l-lysine-coated slides, followed by Shandon Cytospin 3 for 3 min at 1000 rpm. The slides were covered with DAKO before viewing with a confocal laser scanning microscope.

### Analysis of cytochrome *c* localization

BaF3/T315I cells were treated with 1 μM HS-543 for 10 h. To label the mitochondria, the cells were incubated with 100 nM mitochondrion-specific dye (MitoTracker® Red FM; Molecular Probes Inc., Eugene, OR) for 45 min at 37°C prior to fixation. The cells were then suspended on poly-l-lysine-coated slides, followed by Shandon Cytospin 3 for 3 min at 1000 rpm. They were then fixed in 4% PFA for 15 min at room temperature and washed with PBS. The cells were incubated at 4°C overnight with cytochrome *c* antibody (Santa Cruz Biotechnology). After washing twice with PBS, the cells were incubated with mouse fluorescein isothiocyanate (FITC) secondary antibody (Dianova). The cells were also stained with DAPI to visualize the nuclei. The slides were then washed twice with PBS and covered with DAKO before viewing with a confocal laser scanning microscope.

### Tumor xenograft study

All animal experiments were performed in accordance with the guidelines of the INHA Institutional Animal Care and Use Committee (INHA IACUC at Inha University Medical School, under the authority of project number INHA 130318-197. The cells were harvested and mixed in PBS (200 μL/mouse). Six weeks old male BALB/c nude mice (Orient Bio, Korea) were inoculated with 1 × 10^5^ cells in the flank. When the tumor size reached approximately 50-100 mm^3^, they were randomly divided into 4 groups of 5 mice. Then, these mice were given HS-543 or Imatinib by intraperitoneally 5 times a week for 14 days. The treat group was fed with either HS-543 (30 mg/kg and 50 mg/kg) or Imatinib (50 mg/kg), and the control group was fed vehicle. Tumor size and body weight were measured every 2 days. Tumor size was calculated using the formula 0.5 × length × width^2^

### Immunohistochemisry

The tissue sections were blocked with normal goat or horse serum (Vector Laboratories) for 1 h, and incubated at 4°C overnight in 1:50 dilutions of cleaved caspase-3 and proliferating cell nuclear antigen (PCNA) (Cell Signaling Technology). The sections were then incubated with biotinylated secondary antibodies (1:100) for 1 h. The sections were visualized by an avidin–biotin peroxidase complex solution using an ABC kit (Vector Laboratories), which were then washed in PBS and developed with a diaminobenzidine tetrahydrochloride substrate for 15 min and then counterstained with hematoxylin. At least 3 random fields of each section were examined at 400× magnification and analyzed using a computer image analysis system (Media Cybernetics. Rockville, MD).

### Statistical analysis

Data were expressed as the mean ± standard deviation (S.D.). Statistical analysis was performed using ANOVA and unpaired Student's *t-*tests. The differences were considered statistically significant when *p* < 0.01.
